# Association between physiological responses after exercise at low altitude and acute mountain sickness upon ascent is sex-dependent

**DOI:** 10.1186/s40779-020-00283-3

**Published:** 2020-11-05

**Authors:** Yang Shen, Yuan-Qi Yang, Chuan Liu, Jie Yang, Ji-Hang Zhang, Jun Jin, Hu Tan, Fang-Zheng-Yuan Yuan, Jing-Bin Ke, Chun-Yan He, Lai-Ping Zhang, Chen Zhang, Jie Yu, Lan Huang

**Affiliations:** 1 Institute of Cardiovascular Diseases of PLA, the Second Affiliated Hospital, Army Medical University, Chongqing, 400037 China; 2 Department of Cardiology, the Second Affiliated Hospital, Army Medical University, Chongqing, 400037 China

**Keywords:** High altitude, Exercise testing, Sex differences, Acute mountain sickness, Individual susceptibility

## Abstract

**Background:**

Acute mountain sickness (AMS) is the mildest form of acute altitude illnesses, and consists of non-specific symptoms when unacclimatized persons ascend to elevation of ≥2500 m. Risk factors of AMS include: the altitude, individual susceptibility, ascending rate and degree of pre-acclimatization. In the current study, we examined whether physiological response at low altitude could predict the development of AMS.

**Methods:**

A total of 111 healthy adult healthy volunteers participated in this trial; and 99 (67 men and 32 women) completed the entire study protocol. Subjects were asked to complete a 9-min exercise program using a mechanically braked bicycle ergometer at low altitude (500 m). Heart rate, blood pressure (BP) and pulse oxygen saturation (SpO_2_) were recorded prior to and during the last minute of exercise. The ascent from 500 m to 4100 m was completed in 2 days. AMS was defined as ≥3 points in a 4-item Lake Louise Score, with at least one point from headache wat 6–8 h after the ascent.

**Results:**

Among the 99 assessable subjects, 47 (23 men and 24 women) developed AMS at 4100 m. In comparison to the subjects without AMS, those who developed AMS had lower proportion of men (48.9% vs. 84.6%, *P* < 0.001), height (168.4 ± 5.9 vs. 171.3 ± 6.1 cm, *P* = 0.019), weight (62.0 ± 10.0 vs. 66.7 ± 8.6 kg, *P* = 0.014) and proportion of smokers (23.4% vs. 51.9%, *P* = 0.004). Multivariate regression analysis revealed the following independent risks for AMS: female sex (odds ratio (*OR*) =6.32, *P* < 0.001), SpO_2_ change upon exercise at low altitude (*OR* = 0.63, *P* = 0.002) and systolic BP change after the ascent (*OR* = 0.96, *P* = 0.029). Women had larger reduction in SpO_2_ after the ascent, higher AMS percentage and absolute AMS score. Larger reduction of SpO_2_ after exercise was associated with both AMS incidence (*P* = 0.001) and AMS score (*P* < 0.001) in men but not in women.

**Conclusions:**

Larger SpO_2_ reduction after exercise at low altitude was an independent risk for AMS upon ascent. Such an association was more robust in men than in women.

**Trial registration:**

Chinese Clinical Trial Registration, ChiCTR1900025728. Registered 6 September 2019.

## Background

Acute mountain sickness (AMS) is the mildest form of acute altitude illnesses that typically occur in unacclimatized persons upon ascent to elevation at ≥2500 m. AMS consists of a series of non-specific symptoms, including headache, dizziness, lightheadedness, gastrointestinal symptoms and fatigue [1, 2]. Over 50% of individuals develop AMS when ascending to elevation at ≥6000 m [3]. In most cases, AMS spontaneously resolve after a few days at high altitude, but may progress to fatal high-altitude cerebral edema (HACE) [4].

Known risk factors for AMS include ascending speed, arrival elevation and individual susceptibility [3]. People who live at sea level for generations and those with a history of AMS or migraine are also reported to be at high risk for developing AMS [5]. Age, sex, smoking status and obesity have been associated with AMS in some but not all studies [5–9]. For example, younger subjects were found to be more susceptible to AMS [5, 8]. Gonggalanzi et al. [6] also found that age below 55 years was an independent AMS risk factor, but smoking reduced the risk of AMS. In a study by Meier et al. [7], younger age (< 50 years) was an AMS risk factor but smoking was not associated with AMS. Sex discrepancy, but again with controversial results. Lower susceptibility has been reported in men by some studies [10–12], whereas other studies reported either no difference or increased susceptibility in men [13–15].

Upon ascending to high altitude, a number of physiological responses are activated to adapt to decreased arterial oxygen saturation (SaO_2_). Sympathetic autonomic system is activated, with resulting vasoconstriction and increased blood pressure (BP) and heart rate (HR) [16]. Previous studies suggested that SpO_2_ reduction and physiological response after exercise at high altitude could be useful in assessing the degree of acclimatization to high altitude [17, 18]. More specifically, SpO_2_ reduction after exercise prior to ascending has been shown to be a risk factor for severe high-altitude illness (HAI) that included severe AMS, HACE and high-altitude pulmonary edema [8]. However, another study indicated that association between SpO_2_ and AMS is not strongly altitude-independent during the first 7 days of trekking [19]. Another important caveat that adds to the complexity of the controversy is the physiological and functional differences between men and women [20, 21].

In the current prospective cohort study, we examined reduction of SpO_2_ as well as HR and BP changes upon exercise in a group of healthy volunteers prior to ascending from 500 to 4100 m. Characteristics of those who developed AMS vs not were compared. Multivariate analysis was used to determine whether exercise-induced responses at low altitude prior to ascent could be used to predict AMS and whether such an association is sex-dependent.

## Methods

### Design and participants

We performed this prospective cohort study on the Qinghai-Tibet plateau in June 2019. A total of 111 unrelated healthy Chinese Han volunteers born and permanently lived in low altitude (≤500 m) without travelling to high-altitude areas (≥2500 m) in the past 6 months were approached. Exclusion criteria included: a history of AMS, migraine, cardiopulmonary diseases, neurological diseases, psychiatric disorders that prevented the completion of data collection, cerebral vascular diseases, cancer, or liver or kidney dysfunction, long-term use of any medications. Body mass index (BMI) was calculated as body weight in kg divided by square height in meter.

### Exercise program

The testing was conducted prior to the ascent at 500 m. Exercise testing was conducted using a mechanically braked bicycle ergometer (Ergoline 900EL, Ergoline Company, Germany) [22]. The session consisted of a 3-min warm-up period with no resistance, a 3-min initial exercise phase at 25-W workload and a 3-min maintenance exercise phase with 50-W workload. HR, BP and SpO_2_ were recorded during the last minute of the session. BP and HR were recorded using an electronic sphygmomanometer (Omron HEM-6200, Japan). SpO_2_ values was determined using a pulse oximeter (Nonin ONYX OR9500, USA). The average of 3 measures was used in data analysis.

### Assessment of AMS

Subjects ascended from 500 m to 4100 m in 2 days. AMS was assessed using the latest Lake Louise questionnaire [1] at 6–8 h after arriving at 4100 m. Participant completed a 4-item questionnaire with the assistance of an experienced doctor. The items included headache, dizziness or lightheadedness, gastrointestinal symptoms and fatigue. The score for each item ranged from 0 to 3: 0 for no, 1 for mild, 2 for moderate, and 3 for severe. AMS was defined as the total scores at ≥3 points, with at least one point from headache.

### Statistical analysis

Statistical analyses were performed using SPSS 24.0 (Chicago, USA). Continuous variables are expressed as the mean ± standard deviation (SD), and group comparison was conducted using Student’s *t*-test or Welch’s test. Categorical variables are expressed as *n* (%) and compared using the chi-square test or Fisher’s exact test. Univariate logistic regression analysis was performed to evaluate the odds ratio (*OR*) with a 95% confidence interval (CI) for the factors associated with AMS. Then, multivariate logistic regression analysis was conducted of the potential risk factors (*P* < 0.1 for enter, and *P* < 0.05 for stay). Linear regression was applied to assess the correlation between the changes in physiological parameters after exercise and AMS score. A two-sided *P* < 0.05 was considered statistically significant.

## Results

### Subjects with vs without AMS

**Fig. 1:**
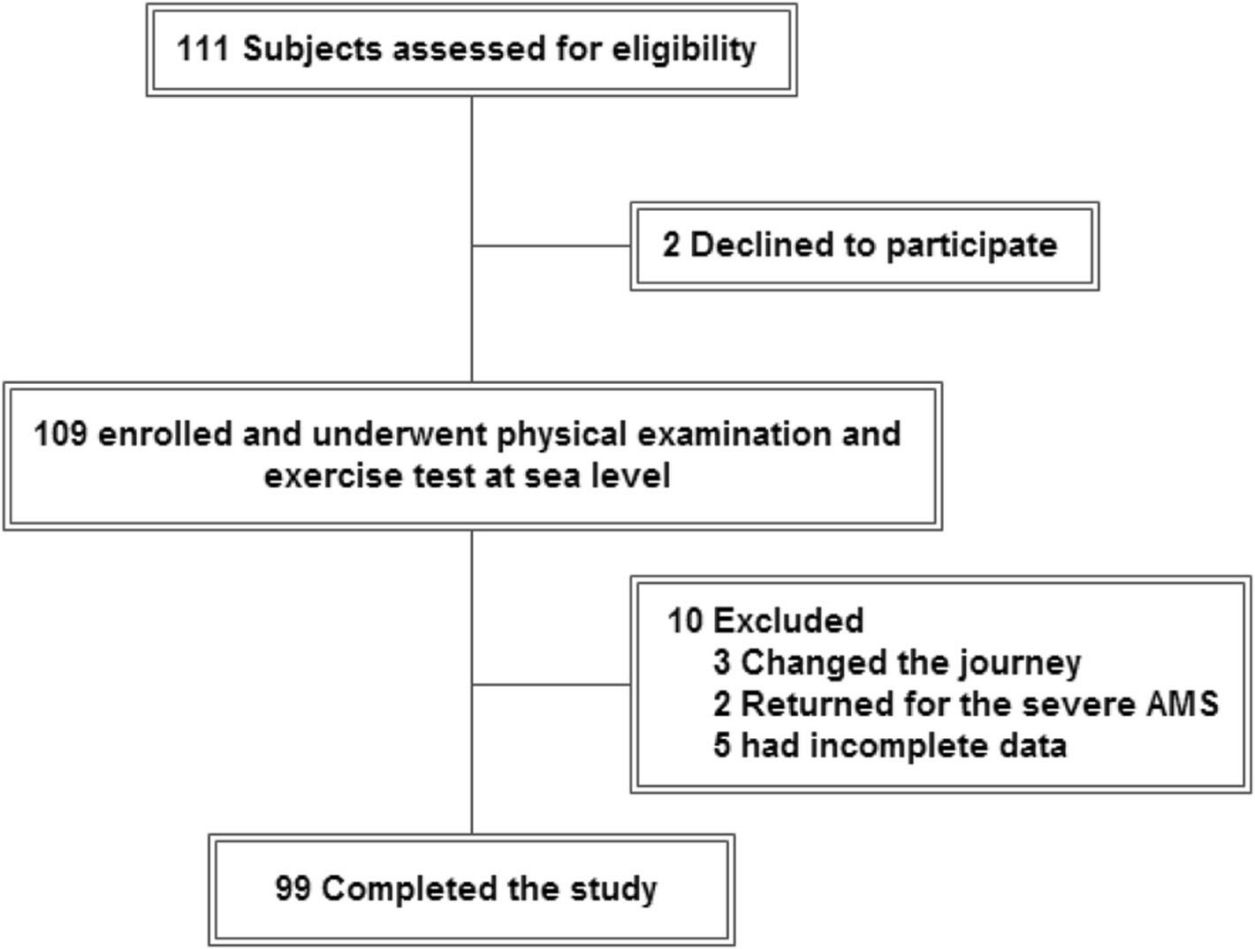
The flowchart of the selection process

A total of 111 healthy adult subjects were invited to participate: 2 refused to participate, 3 did not adhere to the pre-planned ascent plan, 2 developed severe HAI during the ascending process and were immediately transferred to low-altitude areas for emergency medical interventions, and 5 had incomplete data. Among the 99 subjects in the final data analysis, 47 developed AMS (Fig. 1). Age did not differ significantly between the subjects who developed AMS (26.0 ± 7.7 years) vs without AMS (27.6 ± 8.8 years) (Table 1). The AMS group had higher proportion of women (51.1% vs. 15.4%, *P* < 0.001), lower height (168.4 ± 5.9 vs. 171.3 ± 6.1 cm, *P* = 0.019), weight (62.0 ± 10.0 vs. 66.7 ± 8.6 kg, *P* = 0.014) and percentage of smokers (23.4% vs. 51.9%, *P* = 0.004). SpO_2_ at rest at low altitude was higher in the AMS group (97.5% ± 1.2% vs. 96.8% ± 1.4%, *P* = 0.017). SpO_2_ reduction at the end of the 9-min exercise session at low altitude was larger in the AMS group (− 0.6% ± 1.7% vs. 0.4% ± 1.7%; *P* = 0.004). The AMS group also had lower systolic BP (118.2 ± 11.8 vs. 126.0 ± 16.3) mmHg, *P* = 0.008) as well as smaller change in systolic BP (1.0 ± 15.0 vs. 8.7 ± 15.0 mmHg, *P* = 0.013) upon arrival at 4100 m.

**Table 1:** Baseline characteristics, exercise testing results, and measures upon arriving at 4100 m

Index	Total (*n* = 99)	AMS (*n* = 47)	Non-AMS (*n* = 52)	*P*-value
Baseline characteristics				
Age (year, *x* ± *s*)	26.9 ± 8.3	26.0 ± 7.7	27.6 ± 8.8	0.358
Men [*n*(%)]	67 (67.7)	23 (48.9)	44 (84.6)	< 0.001
Women [*n*(%)]	32 (32.3)	24 (51.1)	8 (15.4)	< 0.001
Height (cm, *x* ± *s*)	170.0 ± 6.1	168.4 ± 5.9	171.3 ± 6.1	0.019
Weight (kg, *x* ± *s*)	64.5 ± 9.5	62.0 ± 10.0	66.7 ± 8.6	0.014
BMI (kg/m^2^, *x* ± *s*)	22.3 ± 2.5	21.8 ± 2.7	22.7 ± 2.2	0.070
Smoker [*n*(%)]	38 (38.4)	11 (23.4)	27 (51.9)	0.004
HR at rest (beats/min, *x* ± *s*)	73.2 ± 11.2	74.5 ± 10.9	71.9 ± 11.5	0.257
SpO_2_ at rest (%, *x* ± *s*)	97.1 ± 1.3	97.5 ± 1.2	96.8 ± 1.4	0.017
SBP at rest (mmHg, *x* ± *s*)	117.3 ± 12.1	117.2 ± 12.6	117.4 ± 11.8	0.937
DBP at rest (mmHg, *x* ± *s*)	73.5 ± 11.4	73.8 ± 12.3	73.4 ± 10.6	0.858
After exercise testing				
SpO_2_ after exercise (%, *x* ± *s*)	97.1 ± 1.2	96.9 ± 1.3	97.2 ± 1.1	0.130
ΔeSpO_2_ (%, *x* ± *s*)	−0.1 ± 1.8	−0.6 ± 1.7	0.4 ± 1.7	0.004
After arriving at 4100 m				
SBP at HA (mmHg, *x* ± *s*)	122.3 ± 14.8	118.2 ± 11.8	126.0 ± 16.3	0.008
ΔhSBP (mmHg, *x* ± *s*)	5.0 ± 15.4	1.0 ± 15.0	8.7 ± 15.0	0.013

*AMS* Acute mountain sickness, *BMI* Body mass index, *HR* Heart rate, *SpO*_*2*_ Pulse oxygen saturation, *SBP* Systolic blood pressure, *DBP* Diastolic blood pressure, *Δe* Change after exercise testing (from the pre-exercise level), *Δh* Change after arriving at 4100 m (from the 500-m level)

### Factors associated with AMS

In the univariate analysis that included all subjects, female sex (*OR* = 5.74, 95% CI 2.23–14.78, *P* < 0.001) and higher SpO_2_ at rest at 500 m (*OR =* 1.47, 95% CI 1.06–2.03, *P* = 0.021) were associated with increased risk of AMS. Greater height (*OR =* 0.92, 95% CI 0.86–0.99, *P* = 0.023), heavier weight (*OR =* 0.95, 95% CI 0.90–0.99, *P* = 0.016), smoking (*OR =* 0.28, 95% CI 0.12–0.67, *P* = 0.004) and greater change in SBP after arriving at 4100 m (*OR =* 0.97, 95% CI 0.94–0.99, *P* = 0.016) were associated with decreased incidence of AMS. Multivariate regression that included all subjects identified the following risks for AMS: female sex (*P* < 0.001), greater SpO_2_ reduction after exercise at 500 m (*P* = 0.002) and smaller change in SBP after arriving at 4100 m (*P* = 0.029) (Table 2).

**Table 2:** Regression analyses of the risk for AMS in the entire cohort

Variable	Univariate analysis		Multivariate analysis	
	*OR* (95% CI)	*P*-value	*OR* (95% CI)	*P*-value
Age	0.98 (0.93–1.03)	0.361	Not entered	
Female sex	5.74 (2.23–14.78)	< 0.001	6.32 (2.25–17.74)	< 0.001
Height	0.92 (0.86–0.99)	0.023	–	
Weight	0.95 (0.90–0.99)	0.016	–	
Smoking	0.28 (0.12–0.67)	0.004	–	
HR at rest	1.02 (0.99–1.06)	0.255	Not entered	
SpO_2_ at rest	1.47 (1.06–2.03)	0.021	–	
SBP at rest	1.00 (0.97–1.03)	0.936	Not entered	
DBP at rest	1.00 (0.97–1.04)	0.856	Not entered	
ΔeHR	0.98 (0.95–1.01)	0.231	Not entered	
ΔeSpO_2_	0.83 (0.67–1.03)	0.091	0.63 (0.47–0.84)	0.002
ΔeSBP	1.00 (0.97–1.02)	0.778	Not entered	
ΔeDBP	1.00 (0.97–1.03)	0.982	Not entered	
ΔhHR	1.00 (0.97–1.03)	0.893	Not entered	
ΔhSpO_2_	0.97 (0.88–1.07)	0.525	Not entered	
ΔhSBP	0.97 (0.94–0.99)	0.016	0.96 (0.93–1.00)	0.029
ΔhDBP	0.97 (0.94–1.00)	0.059	–	

*OR* Odds ratio, *95% CI* 95% confidence intervals, *AMS* Acute mountain sickness, *BMI* Body mass index, *HR* Heart rate, *SpO*_*2*_ Pulse oxygen saturation, *SBP* Systolic blood pressure, *DBP* Diastolic blood pressure, *Δe* Change after exercise testing, *Δh* Change after arriving at 4100 m

### Sex discrepancy

Table 3 shows basic characteristics, exercise testing results and measures after arriving at 4100 m in men vs women. In comparison to men, the women were younger (23.3 ± 3.7 vs. 28.5 ± 9.3 years, *P* < 0.001), shorter (165.0 ± 4.1 vs. 172.5 ± 5.2 cm, *P* < 0.001), and lighter (55.4 ± 5.3 vs. 68.8 ± 7.9 kg, *P* < 0.001), had lower BMI (20.5 ± 1.9 vs. 23.1 ± 2.2 kg/m^2^, *P* < 0.001), smaller proportion of smokers (3.1% vs. 55.2%, *P* < 0.001), lower SBP at rest at 500 m (112.7 ± 11.9 vs. 119.4 ± 11.7 mmHg, *P* = 0.011), but higher SpO_2_ at rest at 500 m (97.6 ± 1.2% vs. 96.9 ± 1.4%, *P* = 0.016). After exercise testing, women had lower SBP (118.1 ± 11.7 vs. 126.5 ± 11.4 mmHg, *P* = 0.001) and higher SpO_2_ (97.6 ± 0.8% vs. 96.8 ± 1.3%, *P* < 0.001). Upon arriving at 4100 m, women had lower SBP (113.9 ± 11.5 vs. 126.3 ± 14.6 mmHg, *P* < 0.001), lower SpO_2_ (85.9 ± 4.3% vs. 87.8 ± 3.5%, *P* = 0.034) and greater SpO_2_ reduction (− 11.6 ± 4.2% vs. -9.1 ± 3.9%, *P* = 0.005).

**Table 3:** Baseline characteristics, exercise testing results and measures after arriving at 4100 m: men vs women

Index	Women (*n* = 32)	Men (*n* = 67)	*P*-value
Baseline characteristics			
Age (year, *x* ± *s*)	23.3 ± 3.7	28.5 ± 9.3	< 0.001
Height (cm, *x* ± *s*)	165.0 ± 4.1	172.5 ± 5.2	< 0.001
Weight (kg, *x* ± *s*)	55.4 ± 5.3	68.8 ± 7.9	< 0.001
BMI (kg/m^2^, *x* ± *s*)	20.5 ± 1.9	23.1 ± 2.2	< 0.001
Smoker [*n*(%)]	1 (3.1)	37 (55.2)	< 0.001
HR at rest (beats/min, *x* ± *s*)	75.3 ± 10.3	72.2 ± 11.6	0.195
SpO_2_ at rest (%, *x* ± *s*)	97.6 ± 1.2	96.9 ± 1.4	0.016
SBP at rest (mmHg, *x* ± *s*)	112.7 ± 11.9	119.4 ± 11.7	0.011
DBP at rest (mmHg, *x* ± *s*)	72.1 ± 9.5	74.3 ± 12.2	0.374
After exercise testing			
SpO_2_ after exercise (%, *x* ± *s*)	97.6 ± 0.8	96.8 ± 1.3	< 0.001
SBP after exercise (mmHg, *x* ± *s*)	118.1 ± 11.7	126.5 ± 11.4	0.001
ΔeSpO _2_ (%, *x* ± *s*)	0.0 ± 1.3	− 0.1 ± 1.9	0.791
ΔeSBP (mmHg, *x* ± *s*)	5.3 ± 17.1	7.1 ± 12.6	0.575
Upon arriving at 4100 m			
SpO_2_ (%, *x* ± *s*)	85.9 ± 4.3	87.8 ± 3.5	0.034
SBP (mmHg, *x* ± *s*)	113.9 ± 11.5	126.3 ± 14.6	< 0.001
ΔhSpO_2_ (%, *x* ± *s*)	−11.6 ± 4.2	−9.1 ± 3.9	0.005
ΔhSBP (mmHg, *x* ± *s*)	1.2 ± 14.6	6.9 ± 15.5	0.080

*AMS* Acute mountain sickness, *BMI* Body mass index, *HR* Heart rate, *SpO*_*2*_ Pulse oxygen saturation, *SBP* Systolic blood pressure, *DBP* Diastolic blood pressure, *Δe* Change after exercise testing, *Δh* Change after arriving at 4100 m

### The rate of AMS and relevant symptoms in men vs women

In comparison to men, women had higher rate of AMS (75.0% vs. 34.3%, *P* < 0.001), average AMS score (3.4 ± 2.0 vs. 1.9 ± 1.4, *P* < 0.001). The rate of dizziness, gastrointestinal symptoms and fatigue were also higher in women than in men (71.9% vs. 43.3%, *P* = 0.010, 37.5% vs. 11.9%, *P* = 0.006; 90.6% vs. 58.2%, *P* = 0.001; respectively, Table 4).

**Table 4:** The rate of AMS and symptoms in men vs women

Index	Women (*n* = 32)	Men (*n* = 67)	*P*-value
AMS [*n*(%)]	24 (75.0)	23 (34.3)	< 0.001
AMS score (*x* ± *s*)	3.4 ± 2.0	1.9 ± 1.4	< 0.001
Headache [*n*(%)]	25 (78.1)	42 (62.7)	0.169
Dizziness [*n*(%)]	23 (71.9)	29 (43.3)	0.010
Gastrointestinal symptoms [*n*(%)]	12 (37.5)	8 (11.9)	0.006
Fatigue [*n*(%)]	29 (90.6)	39 (58.2)	0.001

*AMS* Acute mountain sickness

### Factors associated with AMS in men vs women

In the analysis that included only women, AMS was associated with ΔSpO_2_ upon arriving at 4100 m (adjusted *OR =* 1.47, 95% CI 1.01 to 2.12, *P* = 0.042), and not any other factors (Table 5). In the analysis that included only men, AMS was associated with ΔSpO_2_ after exercise testing at 500 m (adjusted *OR =* 0.56, 95% CI 0.39 to 0.79, *P* = 0.001).

**Table 5:** Regression analyses in men vs. women

Variable	Unadjusted analysis		Adjusted analysis	
	*OR* (95% CI)	*P*-value	*OR* (95% CI)	*P*-value
Women				
HR at rest	0.98 (0.91–1.07)	0.695	0.98 (0.88–1.08)	0.632
SpO_2_ at rest	1.06 (0.54–2.09)	0.862	1.12 (0.53–2.38)	0.767
SBP at rest	1.06 (0.98–1.15)	0.155	1.08 (0.96–1.20)	0.199
DBP at rest	0.99 (0.91–1.08)	0.879	0.95 (0.85–1.07)	0.416
ΔeHR	1.02 (0.96–1.08)	0.589	1.01 (0.94–1.09)	0.726
ΔeSpO_2_	1.10 (0.59–2.05)	0.758	0.98 (0.47–2.01)	0.946
ΔeSBP	0.98 (0.93–1.03)	0.348	0.98 (0.91–1.04)	0.442
ΔeDBP	0.96 (0.90–1.03)	0.270	0.96 (0.89–1.03)	0.268
ΔhHR	1.00 (0.95–1.06)	0.959	1.00 (0.93–1.07)	0.938
ΔhSpO_2_	1.21 (0.96–1.53)	0.114	1.47 (1.01–2.12)	0.042
ΔhSBP	0.95 (0.88–1.02)	0.132	0.94 (0.87–1.02)	0.150
ΔhDBP	0.96 (0.90–1.02)	0.197	0.95 (0.87–1.04)	0.302
Men				
HR at rest	1.02 (0.98–1.07)	0.310	1.02 (0.97–1.07)	0.362
SpO_2_ at rest	1.45 (0.95–2.20)	0.083	1.47 (0.95–2.28)	0.086
SBP at rest	1.01 (0.96–1.05)	0.795	1.00 (0.96–1.05)	0.924
DBP at rest	1.02 (0.97–1.06)	0.484	1.01 (0.97–1.06)	0.593
ΔeHR	0.96 (0.91–1.01)	0.090	0.96 (0.91–1.01)	0.100
ΔeSpO_2_	0.57 (0.40–0.80)	0.001	0.56 (0.39–0.79)	0.001
ΔeSBP	1.02 (0.97–1.06)	0.477	1.02 (0.97–1.06)	0.444
ΔeDBP	1.01 (0.97–1.04)	0.748	1.01 (0.97–1.04)	0.792
ΔhHR	1.00 (0.96–1.04)	0.969	1.01 (0.97–1.05)	0.822
ΔhSpO_2_	0.97 (0.85–1.10)	0.617	0.98 (0.86–1.13)	0.785
ΔhSBP	0.98 (0.94–1.01)	0.172	0.97 (0.94–1.01)	0.123
ΔhDBP	0.98 (0.94–1.02)	0.260	0.98 (0.94–1.02)	0.290

*Adjusted analysis* Adjusted for age, height, weight, and smoking status. *AMS* Acute mountain sickness, *HR* Heart rate, *SpO*_*2*_ Pulse oxygen saturation, *SBP* Systolic blood pressure, *DBP* Diastolic blood pressure, *Δe* Change after exercise testing, *Δh* Change after arriving at 4100 m

### Association of AMS score with SpO_2_ reduction after exercise at 500 m

**Fig. 2:**
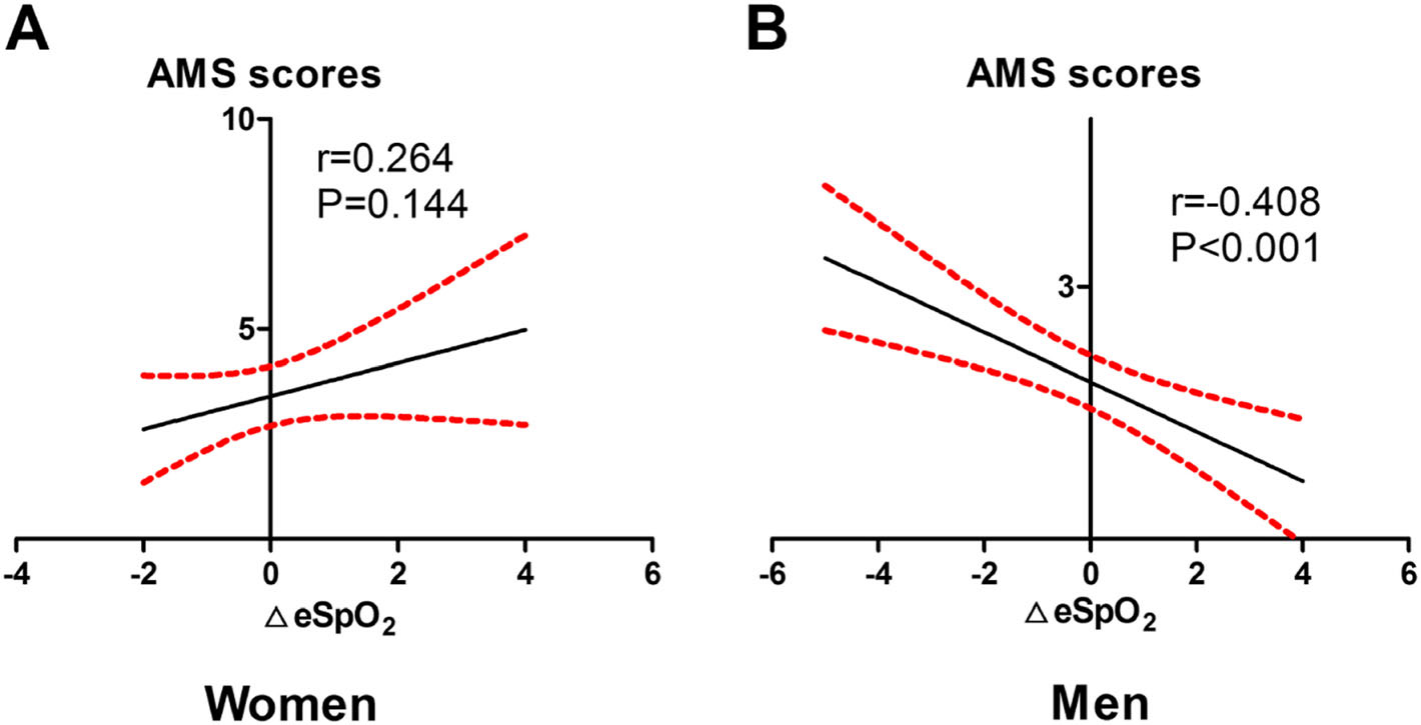
The linear relationship between AMS scores and ΔeSpO_2_ at sea level. The linear relationship between AMS scores and ΔeSpO_2_ in a): Women, b): Men. AMS. Acute mountain sickness; SpO_2_. Pulse oxygen saturation; Δe. Change in value after exercise testing

In the linear regression analysis, AMS score was associated with ΔSpO_2_ after exercise testing at 500 m in men (*r* = − 0.408, *P* < 0.001) but not in women (*r* = 0.264, *P* = 0.144) (Fig. 2).

## Discussion

The results from the current study showed higher AMS rate in women than in men. The female sex and greater SpO_2_ reduction after exercise at low altitude prior to the ascent were independently associated with increased risk of AMS. Interestingly, SpO_2_ reduction at low altitude was associated with the risk of AMS and higher AMS score in men but not in women.

### Incidence of AMS

Previous studies have estimated that 10–70% of travelers will experience various degrees of AMS when ascending to elevation of ≥2500 m [6]. At 4500–5500 m, the incidence of AMS in unacclimatized persons has been estimated at 50–85% [5]. In army recruits ascending from sea level to Lhasa by aircraft, AMS incidence has been reported to be 57% [23]. The rate of AMS at 47.5% in the current study is generally consistent with these previous reports. The discrepancy among the studies may reflect differences in a variety of factors, including field conditions, the speed of ascent, arrival altitude, ethnicity of the enrolled study subjects, as well as timing of AMS assessment after the ascent.

### Sex differences

Sex differences in AMS has been previously report, but with inconsistent conclusions. A meta-analysis supported higher susceptibility in women [24]. In the current study, we confirmed higher rate of AMS in women. Notably, 75.0% of the women in the current study developed AMS upon ascent to 4100 m. This rate is similar to a study by Boos CJ in which 69.2% of women developed AMS [25]. The Boos study also suggested that anxiety at low altitude is an independent predictor of AMS upon ascent, and women tend to have higher level of anxiety. The fact that anxiety levels are higher in younger adults and women may partly explain the difference in AMS susceptibility between women and men [26]. However, Pesce et al. failed to show a difference in the rate of AMS between men and women [27]. Wagner et al. [15] even found higher risk of AMS in men. These inconsistent findings may be attributed to the ethnic and age differences, different levels of anxiety, history of high altitude exposure, experience with hiking, and prophylactic use of pharmacological agents. In addition, Gatterer et al. [28] found that resting cortisol levels at sea level are associated with fluid balance and AMS risk after ascent, suggesting the involvement of autonomic nervous and endocrine system.

Higher incidence of AMS in women may be explained by the effects of hormones. First, testosterone possess potent erythropoiesis action [29]. High serum testosterone and hemoglobin levels are conducive to improvements in oxygen transport, normal cellular function and thus lower susceptibility to AMS. Second, 17 beta-estradiol could reduce the operating point for osmoregulation of arginine vasopressin and contribute to fluid retention [30]. Seventeen beta-estradiol could also up-regulate the expression of vascular endothelial growth factor (VEGF), which in turn promotes endothelial cellular proliferation, angiogenesis and vascular permeability [31]. Fluid retention and increased permeability of the vascular endothelium compromise the blood-brain barrier and promote brain tissue swelling and intracranial hypertension [32].

### Predictive value of exercise testing

Under hypoxia, the sympathetic system is activated to ensure a sufficient oxygen supply. Heart rate variability (HRV) is a common indicator that reflects the balance of cardiac autonomic nervous function between the sympathetic system and the parasympathetic system. HRV has been found to be associated with AMS risk; however, the assessment of HRV requires 12-lead electrocardiogram; more importantly, the prediction value was limited [33]. A more convenient indicator is needed for the general population under field conditions. A previous study suggested that decreased SpO_2_ at rest increases the likelihood of AMS upon ascent to high altitude [34]. Fluid accumulation in the pulmonary vasculature and/or inflammatory reactions in the peripheral airways may reduce pulmonary gas exchange under hypoxic conditions. This may further decrease SaO_2_ and cause hypoxia-induced illness. Exercise testing under hypobaric conditions in laboratory could identify subjects who will develop severe HAI upon ascent in some but not all studies [35, 36]. Also, such method is apparently not suitable as a screening test to identify subjects susceptible for the less severe AMS in the general population [13].

In the current study, SpO_2_ change after arriving at 4100 m was positively correlated with AMS in women. In addition to the effects of hormones, the regulation of the respiratory system and changes in physiological parameters also play important roles in the development of AMS. Relatively smaller tidal volume and higher breathing frequency in women could conceivably lead to increased strain on respiratory muscles under hypoxia and exercise conditions. Women are also more susceptible to hypoxemia, which may explain why the higher incidence of AMS in women observed in the current study as well as in previous studies [37]. We also showed an association between SpO_2_ change after exercise testing at low altitude with AMS risk in the entire cohort, and more so in men. ΔeSpO_2_ after exercise testing at low altitude was also positively correlated with AMS score in the men, suggesting that ΔeSpO_2_ after mild exercise could be a useful tool to predict AMS. Subjects with higher maximal oxygen consumption (VO_2max_) values perform better at endurance exercise. VO_2max_ has been shown to be strongly associated with red cell volume and hemoglobin concentration [38]. Higher VO_2max_, red cell volume and hemoglobin concentration as the result of testosterone stimulation may partly explain the low incidence of AMS in men. When subjects exercised at equal intensity, the oxygen consumption in men was more remarkable, and exercise-induced desaturation could predict AMS risk. However, this did not translate into an increased incidence of AMS in men.

Consistent with a meta-analysis study about smoking and AMS [39], we found a lower percentage of smokers in the AMS group. Smokers have higher basal carbon monoxide (CO) [40], which in turn decrease cerebral blood flow velocities, and thus decreased risk of high altitude headache and AMS [41]. CO could occupy the binding sites of hemoglobin and decrease the oxygen content in the circulating blood [42]. These mechanisms may explain why smokers are less susceptible to AMS in the current study. In a previous study by Wu et al. [43], smoking was also a protective factor against AMS during acute hypoxia exposure. Such a finding by no means advocate smoking, since smoking could impair long-term acclimatization in addition to causing a variety of serious health problems.

### Other factors related to AMS

Age has been inversely associated with AMS in some [44], but not all studies [45]. Trekkers younger than 60 years are twice as likely to develop AMS [46], possibly due to less experience and more rapid ascent. The ratio of cranial cerebrospinal fluid to brain volume increases with age, and may serve as a compensatory adaptation to limit the effect of brain swelling and ultimately decreased susceptibility to AMS. Also, respiratory responses to hypoxia and blood oxygenation increase with age in men, and lung diffusion limitation was less prominent in older people [44]. These findings may help to explain the wide difference in exercise-induced desaturation under hypoxic conditions across age groups.

Ge et al. [47] found higher AMS score and lower SaO_2_ in obese subjects. Such a phenomenon may be partly related to greater SpO_2_ reduction during the night at high altitude. In the current study, BMI was lower in the AMS group. In addition to BMI, however, other factors (e.g., waist, body fat and body composition) may also affect the development of AMS. As a result, BMI should not be considered in isolation. For example, the female sex was strongly associated with increased AMS risk. Whether and how AMS susceptibility in women is connected to lower weight and BMI in women requires further studies.

### Limitations

The present study does have limitations. First, participating subjects were mostly young despite of a wide range (19–59 years). Whether the findings could be extrapolated to older population remains unknown. Second, we used a single elevation and single ascending rate. The findings need to be validated in studies with different protocols. Third, the sample size of women was relatively small (*n* = 32). More importantly, men and women were not well matched by age and BMI. Lastly, the intensity of exercise was mild. SpO_2_ reduction after exercise testing therefore is relatively small. Exercise programs with higher intensity might be more sensitive to identify persons at risk to develop AMS.

## Conclusion

AMS is common if the ascent to 4100 m is completed within 2 days. AMS is more common and severe in women than in men. SpO_2_ reduction at low altitude could be used to predict AMS upon ascent in men but not in women, indicating major sex differences.

## Data Availability

All data generated or analyzed during this study are included in this published article.

## Abbreviations

AMS: Acute mountain sickness
BMI: Body mass index
CI: Confidence intervals
CO: Carbon monoxide
DBP: Diastolic blood pressure
HACE: High-altitude cerebral edema
HAI: High-altitude illness
HIF: Hypoxia inducible factor
HR: Heart rate
*OR*: Odds ratio
SaO_2_: Arterial oxygen saturation
SBP: Systolic blood pressure
SpO_2_: Pulse oxygen saturation
VO_2max_: Maximal oxygen consumption
Δe: Change after the exercise testing at low altitude
Δh: Change after arriving at 4100 m
